# The Canadian Dementia Imaging Protocol: Harmonization validity for morphometry measurements

**DOI:** 10.1016/j.nicl.2019.101943

**Published:** 2019-07-18

**Authors:** Olivier Potvin, Isabelle Chouinard, Louis Dieumegarde, Robert Bartha, Pierre Bellec, D. Louis Collins, Maxime Descoteaux, Rick Hoge, Joel Ramirez, Christopher J.M. Scott, Eric E. Smith, Stephen C. Strother, Sandra E. Black, Simon Duchesne, the CIMA-Q group, the CCNA group

**Affiliations:** a CERVO Research Center, Institut universitaire en santé mentale de Québec, Québec, Canada; b Département de radiologie et de médecine nucléaire, Université Laval, Québec, Canada; c Robarts Research Institute, Medical Biophysics, Western University, London, Canada; d Département de psychologie, Université de Montréal, Montréal, Québec, Canada; e McConnell Brain imaging Center, Montreal Neurological Institute, McGill University, Montréal, Canada; f Université de Sherbrooke, Sherbrooke, Québec, Canada; g LC Campbell Cognitive Neurology Research, Sunnybrook Research Institute, University of Toronto, Toronto, Canada; h Department of Clinical Neurosciences and Hotchkiss Brain Institute, University of Calgary, Calgary, Canada; i Rotman Research Institute, Baycrest Medical Biophysics, University of Toronto, Toronto, Canada

**Keywords:** Neuroimaging, Magnetic resonance imaging, Standardization, Morphometry, Multi-centric studies

## Abstract

The harmonized Canadian Dementia Imaging Protocol (CDIP) has been developed to suit the needs of a number of co-occurring Canadian studies collecting data on brain changes across adulthood and neurodegeneration. In this study, we verify the impact of CDIP parameters compliance on total brain volume variance using 86 scans of the same individual acquired on various scanners. Data included planned data collection acquired within the Consortium pour l'identification précoce de la maladie Alzheimer - Québec (CIMA-Q) and Canadian Consortium on Neurodegeneration in Aging (CCNA) studies, as well as opportunistic data collection from various protocols. For images acquired from Philips scanners, lower variance in brain volumes were observed when the stated CDIP resolution was set. For images acquired from GE scanners, lower variance in brain volumes were noticed when TE/TR values were within 5% of the CDIP protocol, compared to values farther from that criteria. Together, these results suggest that a harmonized protocol like the CDIP may help to reduce neuromorphometric measurement variability in multi-centric studies.

## Highlights

• The Canadian Dementia Imaging Protocol was developed for multi-centric studies.
• This protocol may help reducing morphometric measurement variability.
• The study is based on a large convenience sample.
• A systematic study of the impact of parameter variation remains to be performed.

## 1 Introduction

Quantitative methods to extract specific and sensitive biomarkers from neuroimages (e.g. algorithms, software)(Frisoni et al., 2013) can be shared to ensure that similar post-acquisition processing is performed in a standardized fashion. However, there will be a large variance in the data from laboratory to laboratory if one does not standardize beforehand the process of acquiring the imaging data. This is especially true of longitudinal, multi-centric settings where the wide array of models and vendors, on top of configuration changes throughout the life cycle of these scanners, significantly affects performance and data quality.

To counteract these effects, several initiatives have developed standard operating procedures to minimize variability in neuroimage acquisition. Within the context of aging and neurodegeneration, one of the most well-known of such efforts was advanced by the Alzheimer's Disease Neuroimaging Initiative (**ADNI**)(www.adni-info.org). It has proposed comprehensive cognitive, behavioural, and especially neuroimaging procedures that have been harmonized and implemented across >55 sites in Canada and the U.S. (Jack Jr. et al., 2008).

In Canada, a collection of studies centered on dementias started acquiring data in the 2013–2016 timeframe and needed a common, harmonized process for magnetic resonance imaging (**MRI**) data acquisition that went beyond the ADNI standard of the time. Thus, a group of physicists, physicians and research coordinators developed what is referred to as the Canadian Dementia Imaging Protocol (**CDIP**; www.cdip-pcid.ca)(Duchesne et al., 2019). The CDIP includes the following sequences: (a) an isotropic high-resolution 3D T1-weighted (T1w) MRI; (b) an interleaved PD/T2-weighted MRI; (c) T2* and FLAIR MRIs; (d) a 30+ directions diffusion MRI; and (e) a functional connectivity, resting-state BOLD MRI. Parameters for each one of these sequences were harmonized across three MRI vendors, namely General Electric Healthcare (**GE**), Phillips Medical Systems (**Philips**), and Siemens Healthcare (**Siemens**). Sites are said to be CDIP-compliant when they follow a three-step process, namely qualification of the protocol, on-going quality control using geometric and human phantom scans, and on-going quality assurance during the study.

Yet while harmonization might be appealing in principle, the demonstration of its usefulness remains to be completed. Limited data exists on the pre/post advantages of harmonizing acquisition parameters. This study clarifies some of this uncertainty by assessing the effect of deviation from compliance to the CDIP T1w MRI protocol on brain morphometry variance. Deviation from the CDIP was analyzed for three key parameters (resolution, echo time and repetition time). Our hypothesis was that increasing compliance to CDIP would lower variability in morphometric brain measures, outside of the application of any further specific image pre/post processing. A secondary objective was to release publicly for unrestricted usage this unique dataset, comprising nearly 100 T1w MRI scans of the same individual acquired on various scanners over the course of a few years.

## 2 Materials and methods

### 2.1 Participant

A Single Individual volunteering for Multiple Observations across the Network (SIMON) took part in this study (SIMON Dataset; http://fcon_1000.projects.nitrc.org/indi/retro/SIMON.html SIMON is an ambidextrous male aged between 38 and 46 years old across the scans used in the present study, and was free from known cognitive impairment, neurological disease or psychiatric disorders. The volunteer provided informed consent for all participating studies as well as the release of the MR data.

### 2.2 Participating studies

Several organizations and projects have contributed to the elaboration of the CDIP protocol and the acquisition of SIMON data, namely the *Canadian Alliance for Healthy Hearts and Minds* (Anand et al., 2016)(cahhm.mcmaster.ca); the *Consortium pour l'identification précoce de la maladie d'Alzheimer – Québec* (**CIMA-Q**)(www.cima-q.ca); the O2 study from *the Consortium Québécois sur la Découverte du Médicament* (www.cqdm.org); the *Medical Imaging Trials Network of Canada – C6* (clinicaltrials.gov/ct2/show/NCT02330510); and the *Ontario Brain Institute's Ontario Neurodegenerative Disease Research Initiative* (Farhan et al., 2017)(ondri.ca). CIMA-Q was founded in 2013 with a $2,500,000 grant from the Fonds d'Innovation Pfizer - Fond de Recherche Québec – Santé sur la maladie d'Alzheimer et les maladies apparentées. The main objective is to build a cohort of participants characterized in terms of cognition, neuroimaging and clinical outcomes in order to acquire biological samples allowing 1) to establish early diagnoses of Alzheimer's disease, 2) to provide a well characterized cohort and 3) to identify new therapeutic targets. The principal investigator and director of CIMA-Q is Dr. Sylvie Belleville of the Centre de recherche de l'Institut universitaire de gériatrie de Montréal, CIUSSS Centre-sud-de-l'Île-de-Montréal. CIMA-Q represent a common effort of several researchers from Québec affiliated with Université Laval, McGill University, Université de Montréal, and Université de Sherbrooke. CIMA-Q recruited 350 cognitively healthy participants, with subjective cognitive impairment, mild cognitive impairment, or Alzheimer's disease, between 2013 and 2016.

### 2.3 Participating centers

Most scans were acquired as part of three projects, namely the **CIMA-Q**, the *Canadian Consortium for Neurodegeneration of Aging* (**CCNA**), and courtesy scans at MR manufacturers. Scans were thus obtained at 23 different sites (see also Table 1): The Centre for Addiction and Mental Health (Toronto, Canada), St-Joseph's Healthcare Hamilton (Hamilton, Canada), the MRI unit of the PERFORM Center of Concordia University (Montréal, Canada), the Seaman MRI Center of the University of Calgary (Calgary, Canada), West Coast Medical Imaging – Uptown, the Centre hospitalier universitaire de Montréal (Montréal, Canada), the Centre d'Imagerie Moléculaire de Sherbrooke of the Centre Hospitalier Universitaire de Sherbrooke (Sherbrooke, Canada), Philips (Best, Netherlands), IRM Québec (Québec, Canada), University of British Colombia (Vancouver, Canada), the Brain Imaging Center of the Montreal Neurological Institute (Montréal, Canada), the Hospital for Sick Children (Toronto, Canada), the Unité de Neuroimagerie Fonctionnelle du Centre de recherche de l'Institut universitaire en gériatrie de Montréal (Montréal, Canada), the Centre Hospitalier Universitaire de Montréal (Montréal, Canada), the Brain Imaging Center of the Institut universitaire en santé mentale Douglas (Montréal, Canada), the Ottawa Civic Hospital (Ottawa, Canada), the Peter S. Allen MR Research Center of the University of Alberta (Edmonton, Canada), Queen's University (Kingston, Canada), the Robarts Research Institute at the University of Western Ontario (London, Ontario), the Royal University Hospital Radiology Department (Saskatoon, Canada), Siemens (Erlangen, Germany), the St. Michael's Hospital (Toronto, Canada), the Radiology Department of Sunnybrook Hospital (Toronto, Canada), and the York MRI Facility (Toronto, Canada).

**Table 1:** Characteristics of scans.

Site	Vendor	Model	Resolution (mm^3^)	TE (msec)	TR (msec)	Flip angle	TI (msec)	Plane	Number of scans
CDIP acquisitions									
CAMH	GE	DISCOVERY MR750	1.00	2.94	6.7	11	400	Sagittal	1
Hamilton	GE	DISCOVERY MR750	1.00	3.18	8.2	11	400	Sagittal	1
PERFORM	GE	DISCOVERY MR750	1.00	2.98	6.9	11	400	Sagittal	2^⁎^
Seaman	GE	DISCOVERY MR750	1.00	3.08	7.5	11	400	Sagittal	1
	GE	DISCOVERY MR750	1.00	3.05	7.4	11	400	Sagittal	2
WCMI	GE	SIGNA Pioneer	0.88	3.16	7.4	12	450	Sagittal	2
CHUM	Philips	Achieva	1.00	3.30	7.3	9	ns	Sagittal	2
	Philips	Ingenia	1.00	3.35	7.3	9	ns	Sagittal	1
CHUS	Philips	Ingenia	1.00	3.30	7.3	9	ns	Sagittal	3
	Philips	Ingenia	1.00	3.33	7.3	9	ns	Sagittal	1
IRM Quebec	Philips	Achieva	1.00	3.14	6.9	9	ns	Sagittal	1
	Philips	Achieva	1.00	3.33	7.3	9	ns	Sagittal	2
	Philips	Achieva	1.00	3.31	7.3	9	ns	Sagittal	2
	Philips	Achieva	1.00	3.36	7.4	9	ns	Sagittal	2
	Philips	Achieva	1.00	3.34	7.4	9	ns	Sagittal	1
	Philips	Achieva	1.00	3.35	7.4	9	ns	Sagittal	1
	Philips	Achieva	1.00	3.30	7.3	9	ns	Sagittal	1
UBC	Philips	Intera	1.00	3.28	7.3	9	ns	Sagittal	1
	Philips	Intera	1.00	3.27	7.2	9	ns	Sagittal	1
BIC	Siemens	TrioTim	1.00	2.98	2300.0	9	900	Sagittal	7
	Siemens	Prisma^fit^	1.00	2.96	2300.0	9	900	Sagittal	3
SickKids	Siemens	Prisma^fit^	1.00	2.98	2300.0	9	900	Sagittal	1
IUGM	Siemens	TrioTim	1.00	2.98	2300.0	9	900	Sagittal	7
	Siemens	Prisma^fit^	1.00	2.96	2300.0	9	900	Sagittal	4
	Siemens	Prisma^fit^	1.00	2.98	2300.0	9	900	Sagittal	2
IUSMD	Siemens	TrioTim	1.00	2.98	2300.0	9	900	Sagittal	5
	Siemens	Prisma^fit^	1.00	2.98	2300.0	9	900	Sagittal	1
Ottawa	Siemens	TrioTim	1.00	2.98	2300.0	9	900	Sagittal	1
Peter S. Allen	Siemens	Prisma	1.00	2.98	2300.0	9	900	Sagittal	2
Queens	Siemens	TrioTim	1.00	1.97	2300.0	9	900	Sagittal	1
Robarts	Siemens	Prisma^fit^	1.00	2.98	2300.0	9	900	Sagittal	2
RUH	Siemens	Skyra	1.00	2.98	2300.0	9	900	Sagittal	2
St. Michael's	Siemens	Skyra	1.00	2.98	2300.0	9	900	Sagittal	1
Sunnybrook	Siemens	Prisma	1.00	2.98	2300.0	9	900	Sagittal	1
York	Siemens	Prisma^fit^	1.00	2.98	2300.0	9	900	Sagittal	1
CDIP total									69

Non-CDIP acquisitions									
Philips	Philips	Ingenia CX	1.00	3.30	7.3	9	ns	Sagittal	1
	Philips	Ingenia CX	0.22	5.57	11.8	8	ns	Sagittal	2
IRM Quebec	Philips	Achieva	0.48	na	na	na	ns	Sagittal	2
	Philips	Achieva	0.48	na	na	na	ns	Axial	1
	Philips	Achieva	1.00	3.13	6.9	8	ns	Sagittal	2
	Philips	Achieva	0.69	na	na	na	ns	Sagittal	1
	Philips	Achieva	0.48	na	na	na	ns	Sagittal	1
	Philips	Achieva	1.00	3.14	6.9	8	ns	Sagittal	2
Siemens	Siemens	Prisma	1.00	2.98	2300.0	9	900	Sagittal	1
	Siemens	Prisma	0.58	3.08	2300.0	9	900	Sagittal	2
	Siemens	Skyra	1.00	2.96	2300.0	9	900	Sagittal	1
	Siemens	Skyra	0.58	3.08	2300.0	9	900	Sagittal	1
Non-CDIP total									17

BIC: McConnell Brain Imaging Centre. CAMH: The Centre for Addiction and Mental Health. CHUM: Centre hospitalier universitaire de Montreal. CHUS: Centre hospitalier universitaire de Sherbrooke. IUSMD: Brain Imaging Center of the Institut universitaire en santé mentale Douglas. Hamilton: St-Joseph's Healthcare Hamilton. SickKids: Hospital for Sick Children. IUGM: Institut universitaire de gériatrie de Montréal. Ottawa: The Ottawa Hospital Civic. PERFORM: Concordia University Perform Centre. Peter S. Allen: Peter S. Allen MR Research Centre. Queens: Queens University. Robarts: Robarts Research Institute. RUH: Royal University Hospital. Seaman: Seaman Family MR Research Centre. St. Michael's: St. Michael's Hospital. Sunnybrook: Sunnybrook Research Institute. ns: Not specified. TE: Echo time. TI: Inversion time. TR: Repetition time. UBC: University of British Colombia. WCMI: West Coast Medical Imaging – Uptown. York: York MRI Facility. ^⁎^One scan resolution is actually 0.999 mm^3^.

The MEDICS Laboratory (CERVO Research Center, Québec, Canada) was responsible for MRI data acquisition and analysis including coordination, development and implementation of CDIP at each site; site qualification; site quality control via the single volunteer scans; and on-going quality assurance for the CCNA and CIMA-Q studies.

### 2.4 Image acquisition and processing

For the present study, 86 SIMON scans (nine on GE, 31 on Philips, and 46 on Siemens) were acquired on 28 different 3-Tesla units (10 different models) over the course of eight years from 2009 to 2018 (Table 1). Twenty of these different scanners had more than one scan and most were follow-up scans (64/86) with a mean scan interval of 238 days and a range between 0 and 873 days (GE: 448, 0–823; Philips: 251, 0–873; Siemens: 197, 0–595). The majority (69/86) of these scans were acquired for the CCNA and CIMA-Q studies during development and implementation of the protocol.

The CDIP (www.cdip-pcid.ca) has been previously described elsewhere in detail (Duchesne et al., 2019). Briefly, the T1w acquisition sequence is a sagittal 3D isotropic scan with high resolution (voxel size = 1.0 × 1.0 × 1.0 mm^3^), an acceleration factor of 2 (Siemens: 3D MP-RAGE-iPAT: 2; GE: 3D FAST-SPGR-ASSET 2.0; Philips: 3D TFE-Fast Sense: 2). Parameters for the 3D T1w acquisition were inspired from work done in the ADNI project (Jack Jr. et al., 2008), resulting in an isotropic resolution and the use of accelerated imaging. One should note that even if CDIP calls for precise parameter values, many acquisitions displayed discrepancies with these targets. While this was corrected for future acquisitions (the purpose of having a human phantom being to test compliance and ensuring quality), it provided us with a convenience sample of scans with different parameters. Reasons for non-compliance included human initiative, and reduced precision due to some scanner interfaces not allowing the precise entry and/or import of set values.

Seventeen scans were acquired through other research projects, including visits to manufacturers sites (see Table 1 for more details on parameters). These images form a convenience sample, did not follow any given protocol nor systematically assessed parameter variability, but were acquired in the context of research studies and/or manufacturer visits, with an eye towards research studies. Thus, while these protocols made different technical choices, all of them were deemed suitable for research project purposes, and hence acceptable for our study.

Brain volumes were extracted from each scan using recon-all pipeline of *FreeSurfer* 6.0 (http://freesurfer.net) with default parameters. The total brain volume without the ventricles (brainsegvolnotvent) from the aseg file was used.

### 2.5 Statistical analyses

To verify whether following CDIP parameters reduced morphometric measures variability, we categorized scans based on their parameters (resolution, echo time (TE), repetition time (TR)) in a cumulative fashion of being increasingly compliant with the CDIP protocol: CDIP (scans acquired with parameters within 1% of CDIP values); CDIP±5% (all scans within 5% of CDIP values); CDIP±10% (all scans within 10% of CDIP values); and Any criteria (all scans). For GE and Philips vendors, TR represents the inner loop gradient echo TR, whereas for Siemens it represents the magnetization-prepared rapid gradient-echo (MPRAGE) outer loop TR. Thus, we planned to analyze GE/Philips and Siemens results separately. To compare variance across these categories, *p* values between categories were calculated based on confidence intervals (95%) computed from bootstrapping with 1000 random sampling with replacement. Moreover, to verify the effect of protocol on morphometric measures, we used linear-mixed models with parameter categories as a between factor (each image being within a single category), age as covariate and scanner model as a random factor.

All statistical analyses were conducted in Python using SciPy (Jones et al., 2001) and StatsModels (Seabold and Perktold, 2010) modules.

## 3 Results

### 3.1 Parameter compliance

For unknown reasons, five images from Philips scanners had no TE and TR values in the image headers, and these images were only used for resolution analyses.

As mentioned earlier in Methods, because of the GE scanner interface and human resources training at the visited sites, specific parameter values could not be easily transferred between machines. Thus, even when sites intended to follow the CDIP protocol (9 out of 10 scans), no site matched the aimed CDIP TE value and only one site matched the intended TR value. Further, due to automatic parameter definition, all intended Philips CDIP scans were within 5% of expected TE and TR values.

### 3.2 Impact of parameter compliance on morphometric variance value

**Fig. 1:**
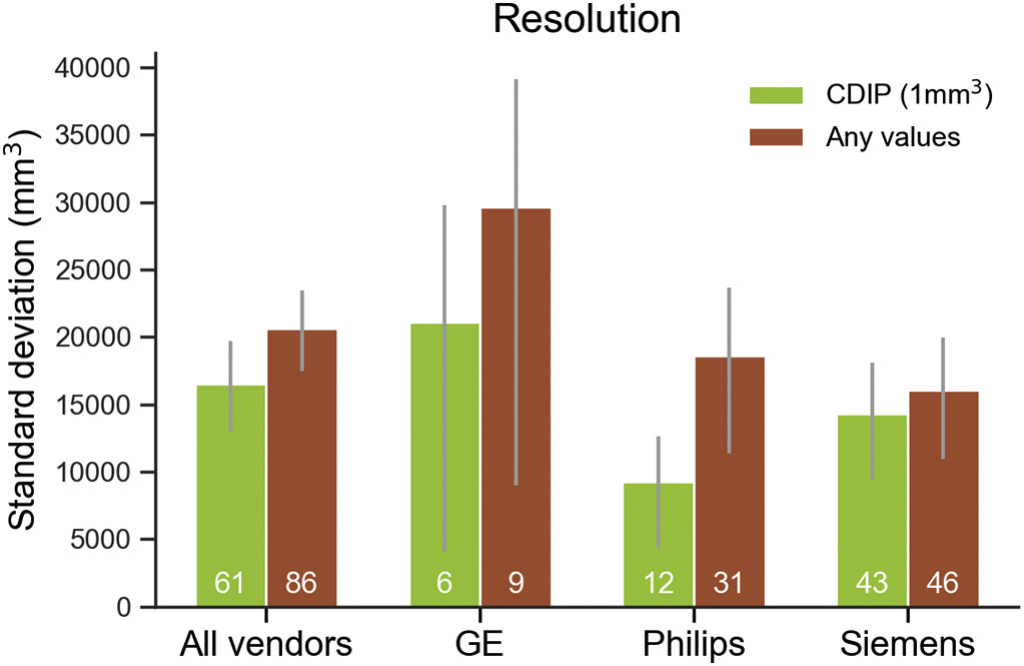
Bar plot showing the median standard deviation of total brain volume across vendors according to cumulative resolution criteria. Error bars denote 95% confidence intervals. White numbers indicate the number of scans meeting each criterion.

**Fig. 2:**
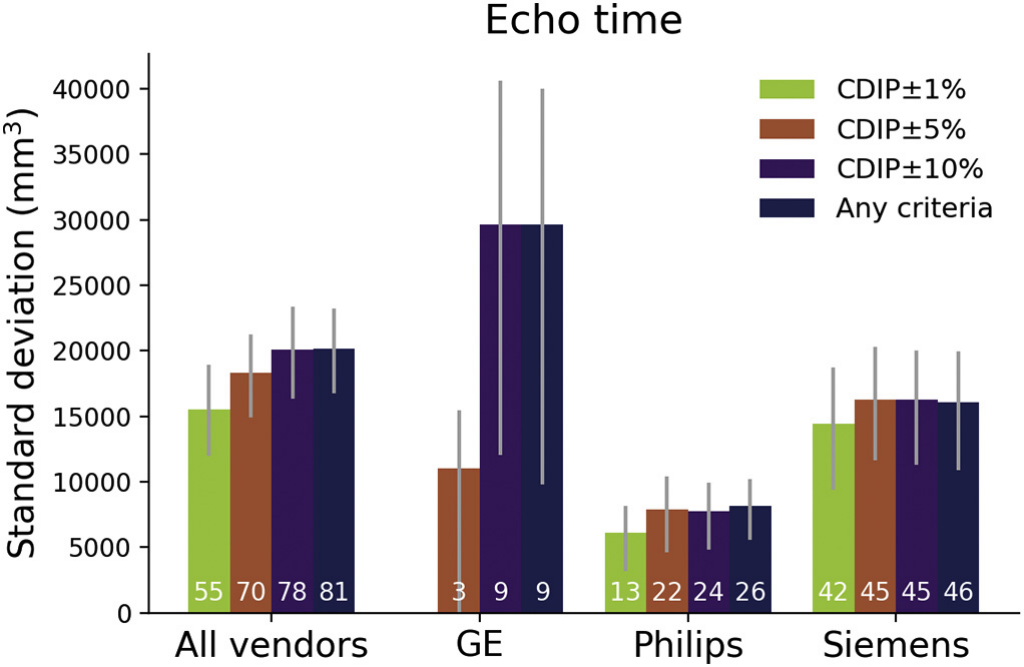
Bar plot showing the median standard deviation of total brain volume across vendors according to cumulative echo time percentage discrepancy from the CDIP criteria (GE 1%: 2.87–2.93 ms, 5% 2.75–3.05 ms, 10%: 2.61–3.19 ms; Philips 1%: 2.75–3.33 ms, 5% 2.83–3.47 ms, 10%: 2.97–3.63 ms; Siemens 1%: 2.95–3.01 ms, 5% 2.83–3.13 ms, 10%: 2.68–3.28 ms). Error bars denote 95% confidence intervals. White numbers indicate the number of scans meeting each criterion.

**Fig. 3:**
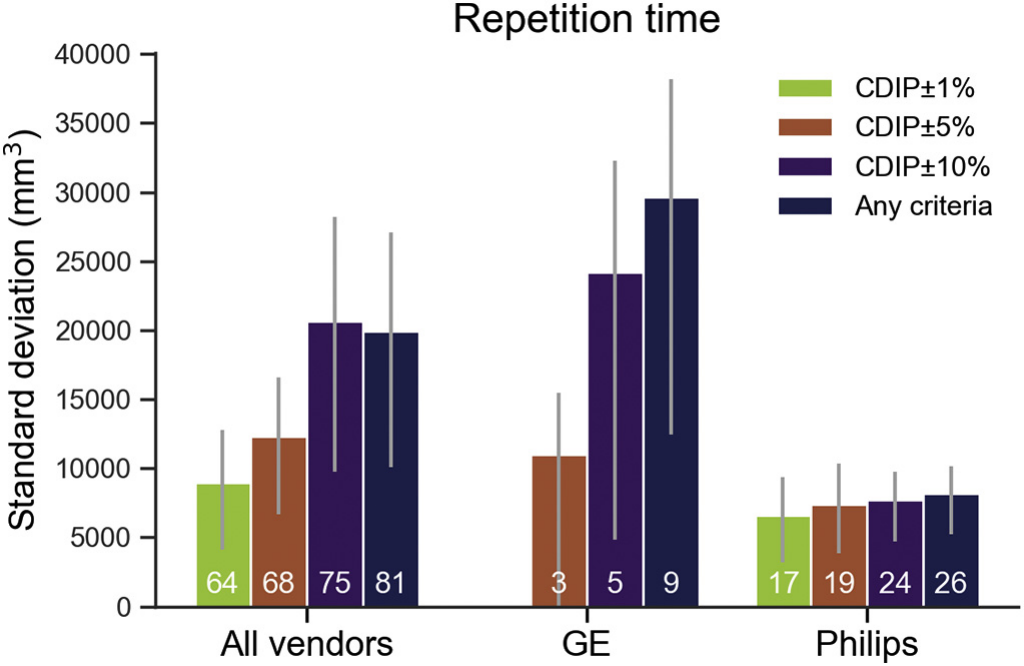
Bar plot showing the median standard deviation of total brain volume for GE and Philips vendors according to cumulative repetition time percentage discrepancy from the CDIP criteria (GE 1%: 6.63–6.77 ms, 5% 6.37–7.04 ms, 10%: 6.03–7.37 ms, Philips 1%: 7.23–7.37 ms, 5%: 6.94–7.67 ms, 10%: 6.57–8.03 ms). Error bars denote 95% confidence intervals. White numbers indicate the number of scans meeting each criterion.

Fig. 1, Fig. 2, Fig. 3 show standard deviations for whole brain volumes across categories for resolution, TE and TR parameters, respectively.

*Resolution:* For resolution, the parameter variability was not sufficient to allow more than two categories, that is either meeting CDIP values (1 × 1 × 1 mm^3^) or not. When all vendors were grouped, the CDIP resolution variance was not significantly lower than the non-CDIP (*p* = .0746). Philips vendor results showed significant lower variance (*p* = .0132) with the CDIP resolution value compared to other values (note that Philips had the largest range of resolutions of all vendors).

*TE:* While variance increased in a stepwise fashion for TE across categories, there was no statistically significant difference between categories. The difference closest to reach significance was between CDIP±1% and both CDIP±10% (*p* = .0664) and “any criteria” (*p* = .0603) categories. Results for GE images indicated significant lower variance with the CDIP±5% compared to the “Any criteria” (*p* = .0220) and CDIP±10% (*p* = .0275) categories.

*TR:* Since there was no variance of TR for Siemens, analyses and Fig. 3 concern only GE and Philips vendors, and thus this study focus on inner-loop TR variability only. Compared to the CDIP±1% category, higher variance was observed with the “Any criteria” category (*p* = .0279). However, this difference was likely due to the GE vendor results since no significant differences between categories were observed for Philips (*ps* > 0.3888) while lower variance with the CDIP±5% compared to the “Any criteria” (*p* = .0133) categories was observed for GE.

For Siemens MRIs, while the interfaces allowed some parameters to be entered directly, the system automatically calculated minimum achievable TE values. Since we presented each unit with the same parameter settings, this calculation showed very little variability between categories and all of them had similar resolution, TE, and TR values.

### 3.3 Impact of parameter compliance on morphometric mean value

**Fig. 4:**
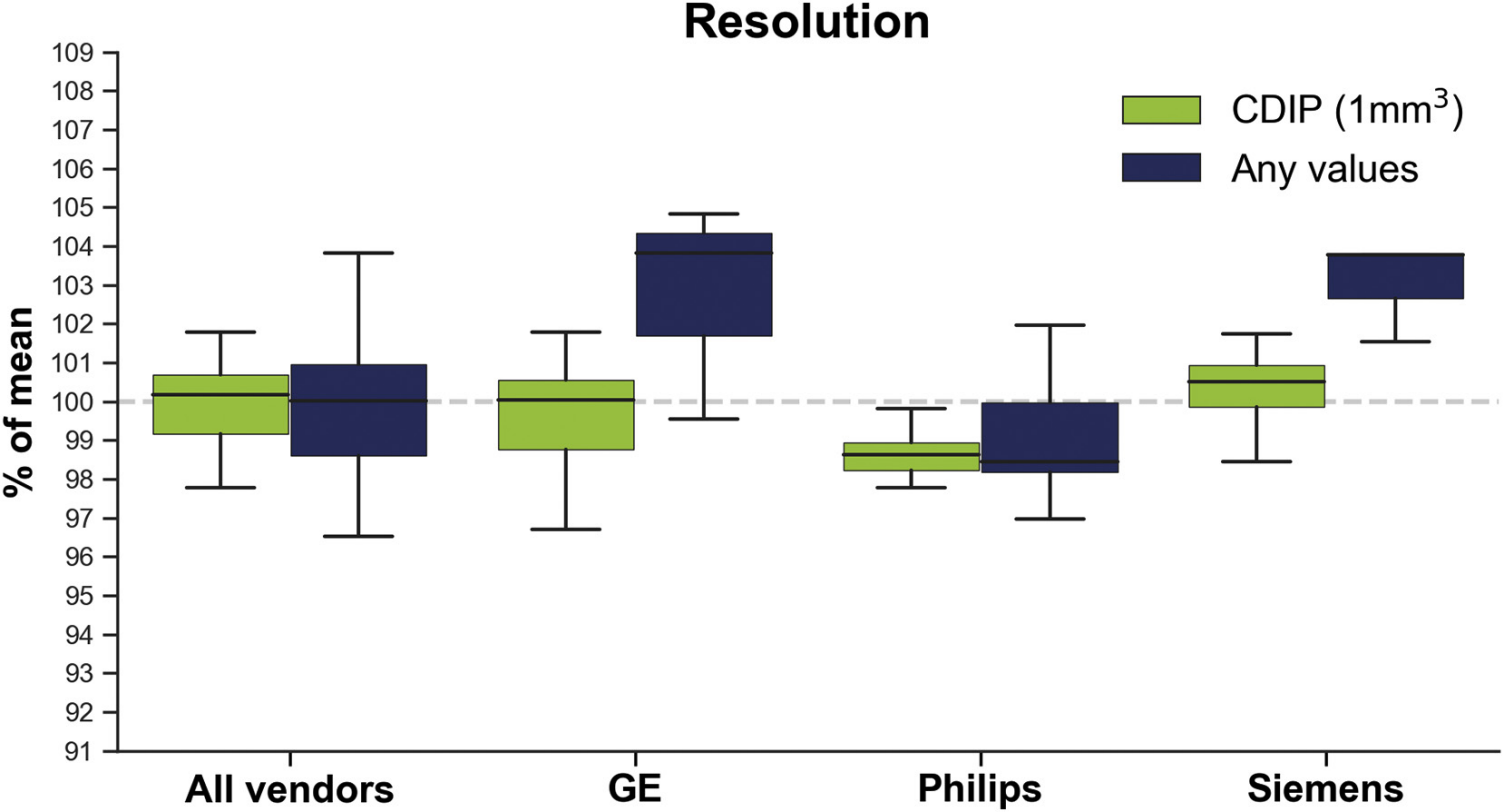
Box plot showing the total brain volume across vendors according to resolution criteria. Boxes show the first and third quartiles with the line denoting the median. Whiskers represent the lowest/highest datum still within 1.5 interquartile range (IQR) of the lower/higher quartile.

**Fig. 5:**
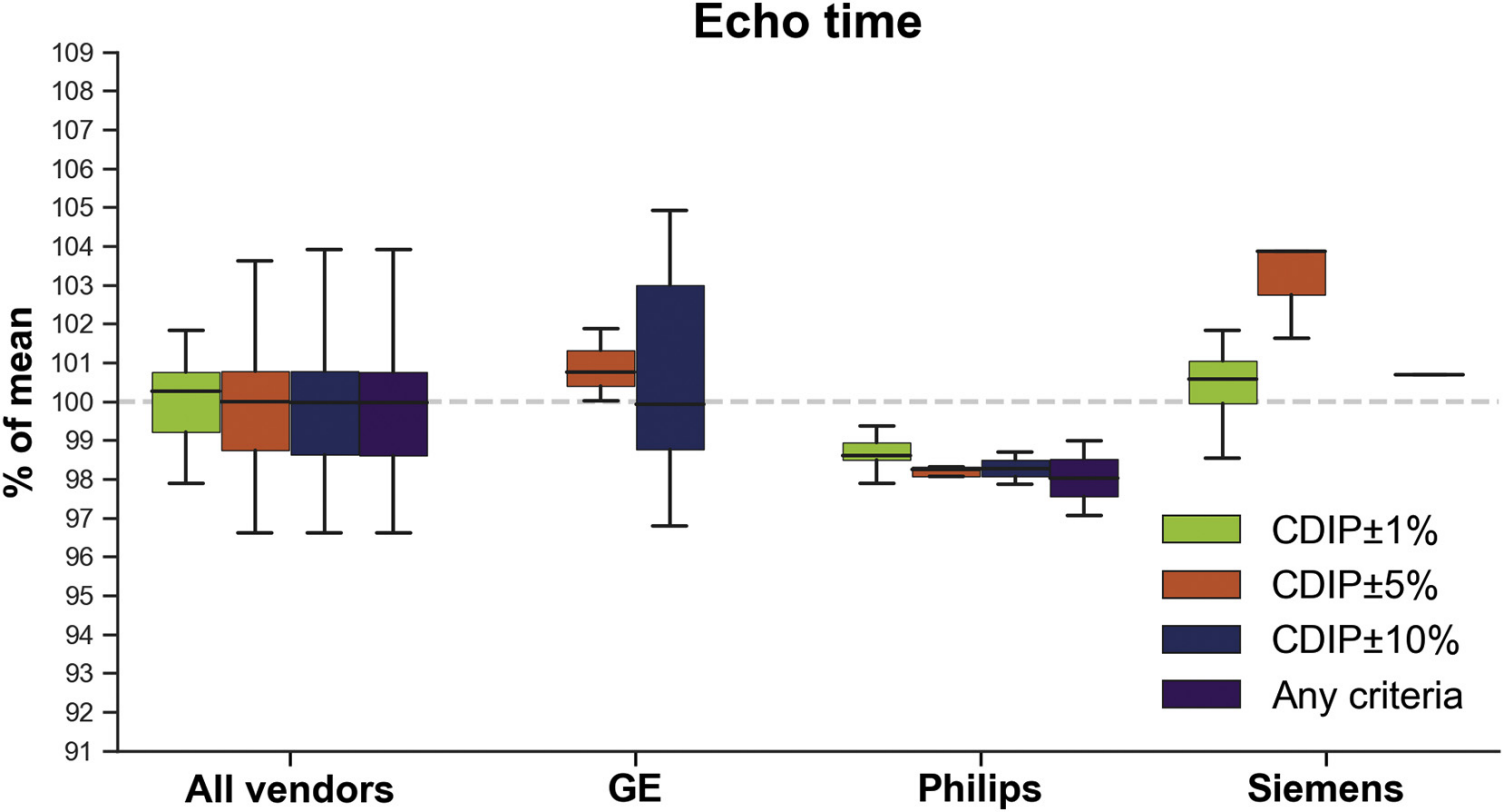
Box plot showing the total brain volume across vendors according to echo time percentage discrepancy from the CDIP criteria (GE 1%: 2.87–2.93 ms, 5% 2.75–3.05 ms, 10%: 2.61–3.19 ms; Philips 1%: 2.75–3.33 ms, 5% 2.83–3.47 ms, 10%: 2.97–3.63 ms; Siemens 1%: 2.95–3.01 ms, 5% 2.83–3.13 ms, 10%: 2.68–3.28 ms). Boxes show the first and third quartiles with the line denoting the median. Whiskers represent the lowest/highest datum still within 1.5 interquartile range (IQR) of the lower/higher quartile.

**Fig. 6:**
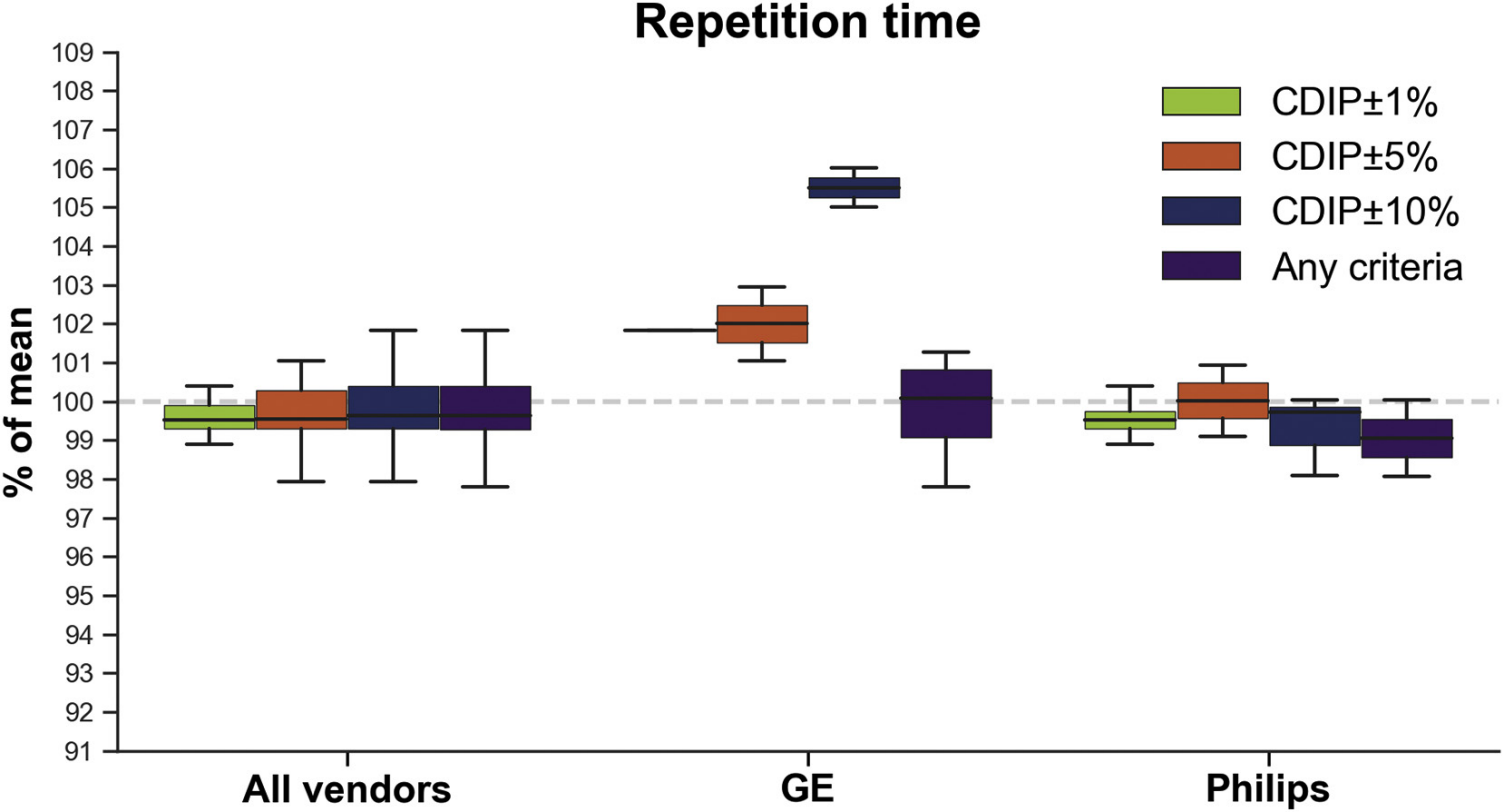
Box plot showing the total brain volume for GE and Philips vendors according to repetition time percentage discrepancy from the CDIP criteria (GE 1%: 6.63–6.77 ms, 5% 6.37–7.04 ms, 10%: 6.03–7.37 ms, Philips 1%: 7.23–7.37 ms, 5%: 6.94–7.67 ms, 10%: 6.57–8.03 ms). Boxes show the first and third quartiles with the line denoting the median. Whiskers represent the lowest/highest datum still within 1.5 interquartile range (IQR) of the lower/higher quartile.

Fig. 4, Fig. 5, Fig. 6 display boxplots showing the median and dispersion of brain volumes across categories for the resolution, TE and TR parameters, respectively. Linear mixed models taking into account age and scanner model, indicated that when all vendors were pooled together, the mean brain volumes were not significantly different between CDIP and non-CDIP resolution values (*p* = .0585). On the other hand, TE CDIP±5% category had significantly larger brain volumes (1.9%, *p* = .0252) compared to the ‘Any criteria’ category and CDIP±1% was also close to significance (1.5%, *p* = .0544). Similarly, for TR (GE and Philips only), larger brain volumes CDIP±1% (1.4%, *p* = .0368) and CDIP±5% (2.0%, *p* = .0047) compared to the ‘Any criteria’ category. Finally, age had a significant effect in the resolution model (*p* = .0003), but not in the TE (*p* = .2094) or TR (*p* = .6574) models, suggesting that TE and TR variations were responsible for that age effect. Because of the lack of parameters variability within each vendor leading to small numbers for many categories, most separated linear mixed models were not possible. Nonetheless, there was a significant effect of resolution for Siemens images, with CDIP images having smaller brain volumes (−2.4%, *p* = .0195).

## 4 Discussion

The harmonized Canadian Dementia Imaging Protocol has been developed to suit the needs of several co-occurring Canadian studies collecting data on brain changes across adulthood and neurodegeneration. In this study, we verified whether using the CDIP was beneficial in lowering variability in gross brain size measurement on a standard post-processing tool, without applying any specific image pre/post processing. We used a substantial convenience dataset of a single individual acquired on 10 scanner models across 86 different sessions, which resulted in many of those not strictly adhering to the CDIP-depicted parameter values.

**Supplementary Fig. 1:**
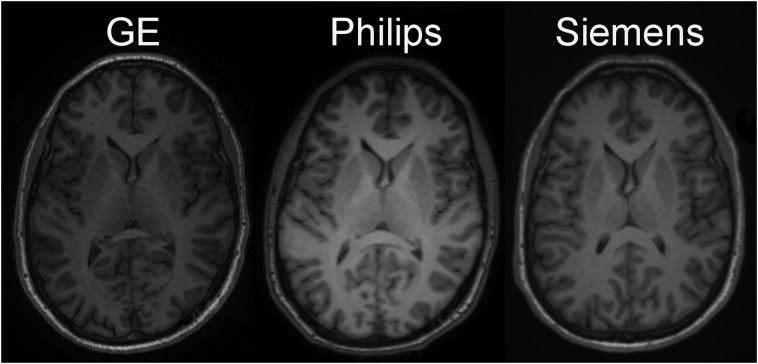
Example of T1 images from GE, Philips and Siemens vendors within 1% deviation of the repetition time value of the CDIP protocol.

First, using the CDIP resolution value (1 × 1 × 1 mm) revealed that compared to other resolution, the CDIP had lower brain volume variance for Philips scanners. One possible factor in explaining the difference in reproducibility between vendors is the choices made during post-acquisition reconstruction of images, for example the application of smoothing/filtering (see Supplementary Fig. 1 for image examples of each vendor). Customization at this processing level is usually not accessible to investigators. Second, brain volume variance appeared to increase as deviation from the CDIP TE and TR parameters increased, but this was significant only for GE scanners. Moreover, some mean effects up to 2% were also observed, notably for the resolution parameter in Siemens scanners, with higher resolution leading to larger volumes compared to the CDIP resolution.

Overall, these findings suggest that increasing non-adherence to a standard protocol, at least for the tested parameters, correspondingly increases morphological variability in brain measurements at a magnitude on par with that of some pathologies of interest – either in a transverse or longitudinal setting. They strengthen the argument for ensuring compliance with a harmonized protocol for multi-centric studies.

Further, the between-vendor offsets could feed into a next iteration of the CDIP protocol, in order to change contrast and bring these measures closer together.

### 4.1 Strengths

One strength of our study is the use of a unique dataset comprising 86 scans of a single individual across multiple scanner models, vendors, and sites. To assess variability across sites in multi-centric studies, such data are required and are relatively rare. The availability of such data across sites and vendors is of paramount importance to the field of image processing. As such, the CDIP group intends to make public all MRI data acquired of SIMON, including all other CDIP contrasts (e.g. T2*, PD/T2w, FLAIR, diffusion and resting state). There have been other instances of similar data that have been acquired and distributed to the community, notably: Brown et al. (2011), who tested 18 participants to investigate sources of variation in the blood oxygen level-dependent signal across four MRI sites; Friedman et al. (2008), who used five participants to estimate the test-retest and between-site reliability of fMRI assessments across 10 MRI sites; Han et al. (2006), who scanned 15 healthy older participants four times at three sites; Suckling et al. (2012) who used 12 participants to carry out a test-retest calibration experiment acquiring functional and structural MRI across three MRI sites; and Suckling et al. (2014) who scanned six participants in order to carry out calibration experiments at each of three MRI sites. Comparatively, our study can contribute a dataset of a single subject, scanned 86 times on 28 different units, which can be useful to answer a number of methodological questions.

## 5 Limitations

The main limitation of our study is that the data originates from a convenience sample, and therefore our acquisitions were not designed to systematically study parameter variability. In fact, it was rather the opposite; the implementation of the protocol was an attempt at removing such variability. However, local variations, interface differences, and a few additional scans – outside of the CDIP project – allowed for the comparisons being presented. In truth, variability mainly differed between vendors due to interface design choices. Sequence implementation choices for some vendors (e.g. Philips, GE) restrict the number of parameters that can be fixed by the user, letting the system automatically calculate values, whereas other manufacturers will allow the complete specification – and therefore transfer – of parameter values from one scanner to another (e.g. Siemens), therefore increasing conformity to a given protocol. One should note that Siemens Healthcare platform is well represented in the Canadian research landscape, it forms the bulk of the images under consideration while the number of GE units is more limited in Canada, resulting in a lower number of images (*n* = 9). Therefore, conclusions regarding GE scanners should be interpreted with caution. Most of our development took place on Philips Medical System scanners, and hence we had the most variability across resolution, TE and TR values for this platform and thus, the strongest conclusions should be drawn for this subsample.

The ideal study would need to compare systematic variations of CDIP parameters. However, such systematic parameters variations would be hard to conduct in a single human subject, especially in a multi-centric context since the same number of scans for each parameters combination would need to be evaluated for each site, yielding a very high number of scans. However, using animals, this type of study could be feasible. For example, a non-human primate could be anaesthetized and scanned with multiple systematic parameters variations, which required to run the sequences multiple times and requires much more time. However, even with this procedure, the logistic of traveling with a live animal, especially in our case (across Canada), would pose other challenges.

Moreover, since our objective was to assess whether the use of the CDIP would be beneficial in lowering the variability in morphometric measures of the brain before applying any specific image pre/post processing, we used the *FreeSurfer* cross-sectional default pipeline to process the images. Thus, we did not aim at maximizing the reliability and other pipelines might yield different variability measurements. Other steps can be done to further minimize variability across sites such as image denoising, registration and distortion correction using geometric phantoms; and in the case of longitudinal data, constrained registration. The use of different pipelines and the impact of these techniques however, extended beyond the scope of this study.

Finally, when comparing images from different manufacturers, one needs to be aware of differences in parameters that may at first pass seem identical. For example, the definition of “sagittal acquisition” is potentially ambiguous, given that there is no real ‘slice select’ direction for acquisition but 2 phase-encoding directions instead, either of which could represent the nominal ‘slice’ direction (and therefore define the nominal orientation). This is important because it will influence the 3D voxel point spread function, and therefore potentially affect the volumetric measurements reported in the manuscript, as well as determining the direction of motion artifacts (e.g. from eye movement).

## 6 Conclusions

The results of the present study suggest that a harmonized protocol like the CDIP may help to reduce variability in neuromorphometric measures for multi-centric studies, but further studies with systematic parameters variation are needed to draw firmer conclusions. The dataset used in this article is available on the International Neuroimaging Data-Sharing initiative platform (INDI; http://fcon_1000.projects.nitrc.org/indi/retro/SIMON.html).

The following is the supplementary data related to this article.
